# The Clarification of Depression and Social Support’s Contribution to the Prediction of Antiretroviral Medication Adherence and the Rate of CD4 in People with HIV

**DOI:** 10.5539/gjhs.v8n9p165

**Published:** 2016-01-22

**Authors:** Zeinab Ebrahimzadeh, Mohammad Ali Goodarzi, Hassan Joulaei

**Affiliations:** 1 Shiraz University, Shiraz, Iran; 2 Departmentof Clinical Psychology, Shiraz University, Shiraz, Iran; 3 Shiraz HIV/AIDS Research Center, Shiraz University of Medical sciences, Shiraz, Iran

**Keywords:** antiretroviral medication adherence, CD4, depression, social support

## Abstract

With the development of the antiretroviral therapy, the number of the people with HIV is increasing; therefore, identifying the factors affecting HIV is of great importance. This study aimed to investigate the relationship between the antiretroviral medication adherence and the rate of CD4 with depression and social support in the people with HIV. The research method was a descriptive study kind of correlation. The statistical population included all patients with HIV in Shiraz, of whom, 220 people who had referred to the Behavioral Diseases Consultation Center were selected using the available sampling method. Philips et al.’s Social Support Questionnaire, Beck’s Depression Questionnaire II, and ACTG Medication Adherence Questionnaire were used as the research tools. Results were analyzed using the stepwise regression and stepwise hierarchical multiple regression. Regression analysis showed that social support and depression variables could predict totally 47% (P<0.001) of changes of medication adherence variable, and depression could predict only 2% (P<0.01) of rate variance of CD4.

## 1. Introduction

Acquired immune deficiency syndrome (AIDS) is a disease caused by human immune deficiency virus, and leads to the severe deficiency of cellular immunity (Kaplan, & Sadock, 2007). This virus undermines the immune system by destroying the immune cells, particularly the cells of CD4 (Mohammadnejadet al., 2009). According to the collected statistics from the universities of medical sciences and treatment services, 27041patients with HIV have been recognized in Iran until September 2013, of whom 89.3% are men and 10.7% women (Ministry of Health, Treatment, and Medical Training; Diseases Management Center, 2013). Antiretroviral treatment has significantly decreased the outbreak of diseases and the mortality rate, caused by HIV (Yaldaet al., 2008). However, medication adherence is rarely considered by many patients. About 50 to 80% of these people may not adhere to the treatment (Belzeret al., 1999; Johnson et al., 2003; Spire et al., 2002). Side effects of the treatment such as nausea or anemia, and some psychological reasons (Vervoortet al., 2007); including the patient’s low rate of self -sufficiency, psychological disorder, depression, trauma, Alzheimer, drug abuse disorders, and low rate of social support are accompanied by weak medication adherence (Ammassariet al., 2002; Deschampset al., 2004; Lesermanet al., 2008).

Social support plays a crucial role in the life of the patient with the chronic diseases. Social support may improve the quality of life in the chronic patients, notably in the patients with HIV (Ncama et al., 2008). Depression is one of the most common secondary complications of HIV, and the most common psychiatric disorders in these people. Depression disorder prevalence among these patients has been reported as 57.3% that is more than five times a normal population (Valente, 2003). Dilorio et al. (2009) evaluated the medication adherence based on the cognitive-social theory. These researchers used a structured model in order to identify the relationship between numbers of psychosocial variables. They reported that lower rate of social support increases the rate of depression, and consequently, reduces the rate of medication adherence (Gardenier et al., 2010). Strong empirical evidences show that social support by friends and family may help the patients to use their medications regularly (Johnson et al., 2010). More signs of depression in patients with HIV is along with lower rate of treatment adherence regimes (Sledjeski et al., 2005; Phillips et al., 2005; Li et al., 2010; Simoniet al., 2002; Avants et al., 2001); and lower rate of CD4 (Sledjeskiet al., 2005). Depression, with no use of anti-depression medications reduces the rate of medication adherence (Yun et al., 2005; Horberg et al., 2008); besides, the higher rate of social support increases the rate of CD4 and the rate of social support among the patients with higher antiretroviral medication adherence is significantly more than that of patients with lower rate of medication adherence (Gardenier et al., 2010).

## 2. Tools and Method

### 2.1 Participants

The statistical population of this study included all the patients with HIV in Shiraz, of whom 220 patients, who had referred to the Behavioral Disorders Consultation Center in Shirazin in Iran, were selected as a sample group using the available sampling method. The patients, who received the antiretroviral treatment, did not suffer from any other physical diseases. They were not affected by the severe psychological disorder or psychotic disorders such as Schizophrenia or mania-depression, and had a very low literacy rate to complete the questionnaire.

### 2.2 Research Tools

Demographic Features: A researcher-developed questionnaire was used for evaluation of demographic features such as age, sex, employment status, marriage status, educational level, duration of the disease, and duration of drug use.

AIDS Clinical Testing Group (ACTG) medication adherence questionnaire: This questionnaire was used for measuring the antiretroviral medication adherence (Chesney et al., 2000). The reliability of the questionnaire was obtained 0.79 using Cranach’s alpha. (Simultaneous) criterion validity of the questionnaire was measured by counting the tablets, which is another method for measuring the treatment adherence rate. Medication adherence measurement by counting the tablets was conducted by referring to the sample group’s medical cases, and calculating the number of the tablets that the patient must had used in the previous four days, and the number of the tablets, which the patient had actually used. Thus, the adherence rate was obtained using the following formula:


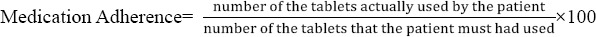


Eventually, Pearson correlation coefficient between the questionnaire’s score and the rate of medication adherence was calculated as 0.82 (p<0.01) by counting the tablet. Beck Depression Inventory (BDI-II): This questionnaire was first introduced by Beck et al. in 1961; It consists of 21 multiple-choice questions which are scored from zero (psychic health) to three (severe depression). Total score’s range is between 0-63 (Groth-Marnat, 2008). This questionnaire was evaluated in Iran for a sample of 354 persons. Its reliability coefficient was obtained by retesting as 0.93 after one week, its convergent validity using Beck Depression Inventory was 0.68, and using the revised measurement of Hamilton psychological scaling for depression, it was reported as 0.71 (Dubson, & Mohammadkhani, 2007).

Social Support Questionnaire: the evaluation scale of social support was developed by Philips, Wax, Helli, Thompson, Williams, and Stewart in 1986. This questionnaire consists of the three domains of family (8 items), friends (7 items), and others (8 items). The range of the questionnaire’s scores is between 0–23 (Ebrahimighavam, 1991). In a study conducted by Mshack (2006), for determination of the questionnaire’s reliability, the test’s total score, and correlated social anxiety test and correlation coefficient was -0.71.

### 2.3 Statistical Method

The research method was a descriptive study kind of correlation. Results were analyzed using the descriptive and inferential statistical methods. SPSS software, correlation, stepwise regression and stepwise hierarchical multiple regression methods were applied. Cronbach’s alpha method was used for evaluating the medication adherence questionnaire’s reliability, and the correlation method was used for measuring its simultaneous tolerance using the tablet count method.

## 3. Results

According to the obtained results, average age of the participants in this study was 38 (N=220) (Table 1). Because most of the studies define adherence rate as using 95% or more of antiretroviral medication, 35.4% of the patients were recognized with medication adherence, and 64.5% with no medication adherence. According to the cutting point of 118 in Beck Depression Inventory, 112 patients (50.9%) showed depression symptoms, and according to the cutting point of 107 in Eshel questionnaire, 42 patients (19.1%) showed the PTSD symptoms.

**Table 1:** Descriptive statistics of the research variables

Feature	Mean	Standard Deviation
Age	38	7.67
Education (Year)	6.08	1.23
Income (Toman)	306770	240540
Disease Duration (month)	70.75	46.7
Duration of drug use (month)	30.1	29.2
Rate of CD4	255.3	154.3
Medication Adherence	14.91	4.07
Depression	20.78	13.84
PTSD	82.80	25.99

Correlation Matrix of demographic features and research variables is shown in Table 2. Results showed that there was a significant negative relationship between age and medication adherences, and between age and the rate of CD4 (respectively, p<0.01, r= -0.20, and p<0.01, r=-0.19), and between disease duration and the rate of CD4 (p<0.05, r=-0.14). There was a significant positive relationship between medication adherence and educational level (p<0.05, r=0.14) and between the rate of CD4 and medication adherence (p<0.01, r=0.20).

**Table 2:** Correlation matrix of demographic features and the variables as the research criteria

Scales	Age	Educational level	Income	Disease Duration	Duration of Drug Usage	CD4 Rate	Medication Adherence
Age	1		-	-	-	-	-
Educational level	-0.12*	1	-	-	-	-	-
Income	0.05	0.28**	1	-	-	-	-
Disease Duration	0.27**	0.03	-0.06	1	-	-	-
Duration of drug use	0.24**	-0.13**	-0.11	0.41**	1	-	-
Rate of CD4	-0.19**	-0.02	-0.02	-0.014*	0.00	1	-
Medication Adherence	-0.20**	0.14**	0.10	-0.01	-0.08	0.20**	1

* P<0.01

** P<0.05.

In this study, there was a significant relationship between age and educational level, and antiretroviral medication adherence; Therefore, stepwise hierarchical multiple regression method was used for clarification of the contribution of two variables of depression and social support to predicting the antiretroviral medication adherence by controlling the variables of age and educational level as the modifier variables. Using the hierarchical regression for predicting the medication adherence rate showed that the effect of the variables in the first stage (age and educational level) was significant only for the age variable (p<0.001). In the second and third stages, respectively, social support and depression variables were inserted in the equation, and with age, 52% of the variations of the medication adherence variable were predicted. Social support was the most effective variable in predicting the medication adherence. Furthermore, there was a significant relationship between age and disease duration, and the rate of CD4; So, in order to predict the rate of CD4 based on depression and social support by controlling the age and disease duration as the modifier variables, stepwise hierarchical multiple regression method was used. Using the hierarchical regression in predicting the rate of CD4 showed that the effect of the variables in the first stage was significant only for the age variable (p<0.004). In the second stage, only depression variable was considered in the equation. It predicted 5% of the variations of CD4 variable with age variable (Table 3).

**Table 3:** Results of multiple regression correlation

Dependent variable	Steps	inserted variables	B	β	t-test		R square	f- test	
	
					T	sig		F	sig
Medication adherence	1^st^ step	age	-0.10	-0.19	-2.91	0.004	0.05	6.80	0.001
		age	-0.09	-0.17	-3.59	0.001			
		Social support	0.56	0.70	14.02	0.001	0.50	74.15	0.001
	2^nd^ step	age	-0.07	-0.13	-2.86	0.005			
		Social support	0.43	0.54	8.19	0.001	0.52	61.99	0.001
	3^rd^ step	depression	-0.07	-0.23	-3.61	0.001			

rate of CD4	1^st^ step	age	-3.26	-016	-2.43	0.004	0.03	5.26	0.004
		age	-2.79	-0.13	-2.00	0.046			
	2^nd^ step	depression	-1.78	-0.16	-2.39	0.017	0.05	5.50	0.001

Because the social support consists of the three domains of family, friends, and others, stepwise multiple regression method was applied in order to determine the role of these dimensions in predicting the medication adherence and rate of CD4. Findings showed that for prediction of antiretroviral medication adherence, regression has proceeded with two steps and variables of family and others have been considered in the equation, and have totally predicted 47% of the variations of medical adherence, however, none of the dimensions of social support could significantly predict the rate ofCD4 in the patients.

**Table 4:** Results of stepwise multiple regression correlation

Dependent variable	steps	inserted variables	B	β	t-test		R square	f-test	
	
					T	sig		F	sig
Medication adherence	1^st^ step	family	1.21	0.65	12.62	0.000	0.42	159.50	0.000
	2^nd^ step	family	0.77	0.41	6.09	0.000	0.47	100.72	0.000
		others	0.74	0.33	4.69	0.000			

## 4. Discussion

Results this study aimed to investigate the relationship between the antiretroviral medication adherence and the rate of CD4 with depression and social support in the people with HIV. Results showed that social support could significantly predict the rate of the antiretroviral medication adherence. Research findings in this regard are consistent with a number of studies (Gardenier et al., 2010; Vyavaharkar et al., 2007; Luszczynska et al., 2007); however, they are not consistent with those of Naurking (2006). According to the Rosenbaum’ self-align model, the lack of enough social support might interfere with cognition alignment process as the basis of medication adherence so that the individual’s self-efficacy expectations are decreased and he concludes that he is not able to change himself, and consequently, he does not expect the desirable results for medication adherence (Godin et al., 2005).

If the patients with HIV receive the enough social support, they can talk to their supporters about the discrimination, ill fame, and problems related to their disease, and may participate in social activities with them. These factors may be the suitable strategies for increasing the medication adherence of the patients.

Findings showed that depression could predict the antiretroviral medication adherence rate. This result is consistent with the previous findings (Li et al., 2010; Phillips et al., 2005; Sledjeski et al., 2005; Yun et al., 2005; Horberg et al., 2008; Vranceanu et al., 2008). In addition, according to the model proposed by Dilorio (2009), there was a direct negative relationship between depression and the medication adherence (Dilorio et al., 2009). This finding showed that depression symptoms may cause the difficulty of the medication adherence for the patients; Low energy, memory problems, and cognitive deficiencies (such as pessimistic thoughts about the effect of antiretroviral medication) that are observed in the depressed people, cause difficulty for medication adherence regimens. Moreover, high suicidal tendencies of the depressed people play a key role in medication non-adherences so that the patients tend to hurt themselves, intentionally. Additionally, severity of depression symptoms could explain almost 3% of CD4 rate variance. This result is consistent with the findings obtained by Sledgesky (2005).

In another study carried out by Horberg (2008), depression treatment was consistent with increase in the rate of CD4. The relationship between depression and medication adherence may be effective in the obtaining these findings. Other depression symptoms include reduction of antiretroviral medication adherence, and probably reduction of medication adherence that reduces the CD4 rate, so that the results of correlation coefficient represent the significant positive relationship between the medication adherence rate and CD4 rate (p<0.01, r = 0.20).

In this study, various aspects of social support (family, friends, and others) were considered in the regression equation in order to predict the antiretroviral medication adherence and CD4 rate. The results of the regression analysis showed that only dimensions of family and others may significantly predict the medication adherence rate among the patients, and the aspect of friends could not predict the medication adherence. None of the aspects of social support could predict the CD4 rate. A study conducted by Li et al. (2010) showed that family relationships had a considerable positive effect on the medication adherence. Family members can remind the patients to take their medications and help them to overcome the side effects of the medications and support them. Findings show that friends’ support could not predict the medication adherence. Therefore, since most of the patients participated in this study were addicted to the drugs, and were infected by HIV through the mutual injection, they stated that they had no more intercommunions with their friends, and even disgusted them. Because their friends were addicts who not only could not support the others, but also they themselves needed others’ support.
